# Group B Streptococcus Infections in Non-Pregnant Adults, Italy, 2015–2019

**DOI:** 10.3390/pathogens13090807

**Published:** 2024-09-18

**Authors:** Monica Imperi, Giovanni Gherardi, Giovanna Alfarone, Roberta Creti

**Affiliations:** Department of Infectious Diseases, Antibiotic Resistance and Special Pathogens Unit, Istituto Superiore di Sanità, 00161 Rome, Italy; monica.imperi@iss.it (M.I.); giovanni.gherardi@iss.it (G.G.); giovanna.alfarone@iss.it (G.A.)

**Keywords:** *Streptococcus agalactiae*, group B streptococci, GBS, adult, infection, molecular epidemiology, antibiotic resistance

## Abstract

Group B Streptococcus (GBS, *Streptococcus agalactiae*) is a pathogen of increasing importance in adults. Severe and invasive cases in non-pregnant adults were collected during the period 2015–2019 by voluntary-based surveillance. In total, 108 GBS strains were phenotypically and genotypically characterized for the serotype, antimicrobial resistance, pili, surface protein genes, and the hyper-virulent adhesin *hvgA*. Patients were divided into two age groups: adults (18–64 years; *n* = 32) and older adults (≥65 years; *n* = 72). The average age was 70.8 years, with a male/female ratio of 1.7. Most isolates were recovered from cases of bacteremia (blood, *n* = 93), and a higher frequency of invasive GBS infections (iGBS) was found among older adults (66.7%). Serotype III was the most frequent (*n* = 41, 38%), followed by type Ia and type V (*n* = 20 each, 18.5%). Serotypes Ia, Ib, II, III, IV, and V accounted for all but one isolates (99.1%). The iGBS isolates were universally susceptible to penicillin, while the prevalence of resistance to clindamycin, erythromycin, tetracycline, and high-level gentamicin resistance was 26.8%, 24.1%, 85.2%, and 5.5%, respectively, with the predominance of the *erm*(B) gene for macrolide resistance and the *tet*(M) gene for tetracycline resistance. The associations between the serotypes/antimicrobial resistance/virulence traits underlined the increasing importance of serotype III and its contribution to antimicrobial resistance as well as the steady increase over time of serotype IV. This nationwide study confirmed the need for monitoring the GBS epidemiology in non-pregnant adults through continuous surveillance of GBS infections.

## 1. Introduction

*Streptococcus agalactiae* or Group B Streptococci (GBS) are Gram-positive bacteria that are part of the normal flora of the gastrointestinal and genitourinary tract of up to 30% of healthy adults [1]. Severe or invasive GBS (iGBS) disease was rarely identified in humans until the 1960s, when increasing numbers of reports were published. Since then, the incidence of iGBS disease has continued to increase and *S. agalactiae* remains a significant pathogen for both infants and adults [2,3,4,5]. In particular, GBS are the leading cause of neonatal and infant invasive infections, causing early-onset disease (0–6 days of age; EOD) and late-onset disease (7–89 days; LOD) [6,7,8,9]. In pregnant women, GBS can cause a variety of illnesses, both during pregnancy and in the post-partum period, ranging from urinary tract infections to chorioamnionitis and sepsis [10]. In non-pregnant adults, the most common clinical manifestations of iGBS disease include bacteremia without a focus and skin/soft tissue infections [11,12,13,14,15,16]. The former often presents with altered mental status, chills, and fever [17]. Bacteremia may also occur secondary to a focal source of infection, in several cases as polymicrobial bacteremia [18,19]. Adult iGBS infections can also result in meningitis, endocarditis, pneumonia, urosepsis, streptococcal toxic shock syndrome, peritonitis, empyema, osteomyelitis, and deep tissue infections [12,16,19,20,21,22,23,24,25,26]. An increase in the incidence of iGBS in adults, especially in older patients (i.e., >65 years), which is associated with higher mortality, has been reported [27,28]. Additional risk factors frequently associated with higher rates of iGBS disease include the Black race, underlying medical conditions such as diabetes mellitus, obesity, liver cirrhosis, cancer, heart and neurological disease, and immunosuppressive conditions [12,19,21,24,29,30].

Intrapartum antibiotic prophylaxis (IAP) or antibiotic treatment is successfully used to prevent and treat GBS infections. Penicillin is the first-line antibiotic; however, concern is increasing about the possible emergence of β-lactam resistance due to occasional reports of GBS with reduced or non-susceptibility to β-lactams, which is associated with mutations in the *pbp2x* gene [31,32,33,34,35]. Erythromycin and Clindamycin are recommended as second-line drugs for patients allergic to β-lactams, but resistance to these antimicrobial agents has long been known and is increasingly being reported worldwide [11,36,37,38,39,40]. The antimicrobial resistance and multidrug resistance (MDR) in GBS highlight the need for a universal GBS vaccine that helps protect not only newborns, infants and pregnant women but also older adults with underlying comorbidities.

GBS produce a polysaccharide capsule characterized by the cell-wall-specific Lancefield’s Group B antigen [41]. To date, ten capsular serotypes have been described in GBS: Ia, Ib and II–IX [17,42]. The capsular-type polysaccharides are important virulence factors and major targets for vaccine formulations currently under development [43]. The capsular serotypes are associated with different invasive potential [44], type of infection, age group and geographic region [45,46]. Serotype III is responsible for the majority of invasive neonatal and infant infections, mainly meningitis, while serotypes Ia, III and V are responsible for 60% of adult iGBS infections worldwide [47,48]. In recent years, the emergence of an MDR serotype III CC17 subclone is a cause for concern [49,50].

In addition to the type-specific polysaccharides, other recognized GBS virulence factors are surface adhesins such as those of the alpha-like protein (Alp) family, pili structures and the hypervirulent HvgA protein [51]. Protein vaccines based on Alp subunits are undergoing clinical trials [52,53,54]. Pili promote bacterial colonization of epithelial cells, support biofilm formation, and facilitate translocation across the blood–brain barrier [55,56,57]. HvgA efficiently supports bacterial adhesion and GBS transfer across the intestinal wall; it also mediates transfer across the blood–brain barrier, specifically the vascular endothelium and the choroid plexus, which are fundamental for promoting meningitis [58].

The aim of our study was to characterize GBS isolates from severe and invasive disease in adults, collected from 2015 to 2019 in Italy, in order to fill a knowledge gap concerning this type of infection in our country and to compare their characteristics with other, similar surveillances. Overall, 108 GBS isolates from a nationwide voluntary-based collection were phenotypically and genotypically characterized for the serotypes, antimicrobial susceptibility profiles and genes encoding pili, Alp surface proteins, and the hyper-virulent adhesin HvgA.

## 2. Materials and Methods

### 2.1. Case Definition

The inclusion criteria for iGBS infections were culture-proven (GBS growth on broth or agar plates) from a normally sterile site (blood and/or cerebrospinal fluid, joint fluid, peritoneal fluid, bone, internal organs) or a clinically severe illness for which no other bacterial etiology has been identified and in which GBS are isolated or detected from a non-sterile site (e.g., wound, superficial skin abscess, lower respiratory tract) [59].

### 2.2. Data Collection

From 2015 to 2019, hospital clinical microbiology laboratories were asked to report iGBS cases and bacterial isolates to the Streptococcal National Reference Laboratory of Istituto Superiore di Sanità (ISS-NRL, Rome, Italy). This study was based on voluntary reporting and is therefore not a population-based survey. A questionnaire, tailored to the adult iGBS surveillance, included anonymous demographic data and clinical information on the site of isolation, type of infection, risk factors, and outcome.

### 2.3. Bacterial Isolates and Serotyping

In cases of duplicate GBS isolates from the same patient and the same site, the first isolate was included in this study. A total of 108 non-redundant GBS isolates were received by the ISS. The bacterial strains were plated on defibrinated sheep blood agar plates and incubated at 37 °C in 5% CO_2_. Identification of colonies as GBS was confirmed by using both Chromatic^TM^ GBS agar plates (Liofilchem, Teramo, Italy) and the Dryspot Streptococcal Grouping Kit (Oxoid, Thermo Fisher Scientific, Monza, Italy). Serotyping (Ia, Ib, II–IX) was performed by the latex agglutination test using the IMMULEX STREP-B Kit (Statens Serum Institute, Copenhagen, Denmark) [60,61,62]. Molecular typing of capsular types Ia–IX was also performed using a multiplex PCR assay for the assignment of non-typeable strains and for confirming the agglutination test [63].

### 2.4. Antimicrobial Susceptibility Testing and Macrolide Resistance Phenotype

Susceptibility testing was performed by E-test gradient strip and/or qualitative disk diffusion according to the European Committee on Antimicrobial Susceptibility Testing (EUCAST) (http://www.eucast.org, last access on 15 August 2024). All the isolates were tested for susceptibility to benzylpenicillin (PEN G), clindamycin (CLI), erythromycin (ERY), tetracycline (TET), and high-level gentamycin resistance (HLGR) using the recently proposed clinical cut-off [64]. The macrolide resistance phenotypes were determined by a double-disk test according to the EUCAST guidelines [65,66]. The isolates were classified as expressing the M-phenotype when they were resistant to macrolides only, or to the MLS_B_ phenotype when showing cross-resistance to macrolides and lincosamides, either constitutive (cMLS_B_ or CR) or inducible (iMLS_B_ or IR).

### 2.5. Bacterial Strain Genotyping

The total bacterial DNA was prepared by a Chelex-based procedure using an InstaGene Matrix (Bio-Rad, Hercules, CA, USA) and treated according to the manufacturer’s instructions.

The presence of the macrolide resistance genes *erm*(A) (subclass *erm*(TR)), *erm*(B) and *mef* (*mef*(A) or *mef*(E)) was investigated in a multiplex PCR, as already described [67,68]. Tetracycline-resistant isolates were screened for the presence of the *tet*(M) and *tet*(O) genes [65,68]. HLGR isolates were assessed for the presence of a complete *aac(6′)-aph(2″)* gene by a PCR assay, as previously described [69]. The presence of GBS alpha (*bca*) and alpha-like (*epsilon*, *rib*, *alp2/3*, and *alp4*) surface protein genes and of the pilus islands PI-1, PI-2a, and PI-2b was detected by PCR [70,71,72,73]. Identification of the hypervirulent ST-17 lineage was performed by using a PCR assay based on the detection of the *hvgA* gene [72].

### 2.6. Statistical Analysis

Comparisons between variables, such as sex, clinical syndrome, isolation site, population group (18–64 years and ≥65 years), serotype, and year of isolation, were statistically measured using the chi-square test, with a *p* value < 0.05 considered statistically significant.

## 3. Results

### 3.1. Patients, Isolates and Clinical Manifestations

From 2015 to 2019, a total of 108 *S. agalactiae* isolates were collected from patients with an average age of 70.8 years (range: 29–97 years). The patients were divided into two subpopulations: adults (18–64 years; *n* = 32/104; 30.8%) and older adults (≥65 years; *n* = 72/104; 69.2%). Age was not reported for four patients. The male/female ratio was 1.7 (65 vs. 38, respectively); gender was not reported for five cases. The male/female ratio showed fluctuations during the study period and was statistically significant in the year 2018 (61.9% female vs. 42.9% male, *p* < 0.05) and in the year 2019 (84.2% male vs. 15.8% female, *p* < 0.05) (Table 1).

iGBS were mainly recovered from cases of bacteremia (blood, *n* = 93), followed by skin and soft tissue infections (*n* = 8), intra-abdominal infections (peritoneal or pelvic fluid, *n* = 2), and pneumonia (bronchial aspirate, *n* = 1) (Table 1). The site of isolation was not available for four GBS isolates (three serotype III and one serotype II). No statistically significant associations were observed between the sex, age group or isolation site (Table 1). Overall, there was a higher frequency of iGBS isolated from the blood among older adults, although not statistically significant (93.1% vs. 80.6%, *p* = 0.094) (Table 1).

### 3.2. Serotype Distribution

Serotype III isolates were the most responsible for the iGBS infections (*n* = 41), followed by type Ia (*n* = 20) and type V (*n* = 20) (Table 2). Overall, serotypes III, Ia, and V accounted for 75.9% of iGBS cases. Capsular types VI, VII and VIII were not detected. No statistically significant associations were found between particular serotypes and the clinical syndrome, age groups or sex (Table 2, *p* values > 0.4).

Fluctuations in the frequencies of the serotypes were observed over the years, but there were no significant variations in their relative proportions throughout the study period, with the exception of serotype IV, which had a statistically significant increase in 2019 (*p* = 0.0478) (Table 3).

### 3.3. Pili and hvgA Distribution

At least one of the three pilus islands (PI) was detected in each GBS isolate (Table 3). The most frequently identified “pilus type” was the combination of PI-1 plus PI-2a (*n* = 48, 44.4%), followed by PI-2a alone (*n* = 28, 25.9%), PI-1 + PI-2b (*n* = 20, 18.5%), and PI-2b only (*n* = 12, 11.1%) (Table 3). The two most frequent PI combinations, although widely distributed in multiple serotypes, were typical of serotypes Ia, Ib, II, and V (Table 3).

The presence of PI-2b, alone or in combination with PI-1, was only observed in serotypes III and IV and was correlated with the presence of *hvgA* (Table 3 and Table 4).

In particular, 29 GBS isolates (25 serotype III and 4 serotype IV) possessed *hvg*A, a marker used to identify the ST-17 clonal lineage in serotype III (Table 3). Of these, seven were attributable to the MDR serotype III CC-17 sub-clone as they possessed pili type 2b only and the *erm*(B) and *tet*(O) genes, as described by Campisi et al. [11] (Table 4).

### 3.4. Antimicrobial Susceptibility Phenotypes and Genotypes

All 108 isolates were susceptible to PEN G, while resistance to CLI, ERY, TET, and HLGR was detected in 26 (26.8%), 29 (24.1%), 92 (85.2%), and 6 (5.5%) isolates, respectively.

Tetracycline resistance was mostly associated with the *tet*(M) gene alone (*n* = 71, 77.2%), but the *tet*(O) gene alone (*n* = 16, 17.4%) and the simultaneous presence of tetM + tetO (*n* = 4; 4.3%) were also found (Table 3). For one iGBS isolate, tetracycline resistance could not be attributed to the *tet*(M) or *tet*(O) gene. The prevalence of erythromycin and clindamycin resistance was 26.8% (*n* = 29) and 24.1% (*n* = 26), respectively (Table 3). Most erythromycin-resistant isolates displayed the CR/*erm*(B) phenotype/genotype (*n* = 20, 69.0%), followed by the IR/erm(A) (subclass erm(TR) (*n* = 6, 20.7%) and M/*mef*A/E (*n* = 3, 10.3%) phenotypes/genotypes (Table 3). Macrolide resistance was associated with serotypes III (*n* = 12), V (*n* = 8), Ib (*n* = 4), Ia (*n* = 3), and IV (*n* = 2) (Table 3). Of particular note was the sharp increase in resistance to erythromycin and clindamycin, which increased from 14.3% and 0%, respectively, in 2015 to 38.1% for both antibiotics in 2019. The prevalence of tetracycline resistance, on the other hand, showed a fluctuating trend over time (Table 5).

HLGR, an emerging resistance in GBS, was identified in four, one, and one isolates of serotype IV, Ib, and V, respectively (Table 3). A total of 25 out of 108 GBS isolates (23.1%) were MDR [74], resistant to tetracycline, erythromycin, and clindamycin (11 serotype III isolates, 7 serotype V isolates, 4 serotype Ib isolates, 2 serotype IV isolates, and 1 serotype Ia isolate); one of these (serotype Ib) was also HLGR (Table 3).

## 4. Discussion

In many parts of the world, an increasing incidence of iGBS infections in adults, compared to neonates and infants, has been reported [15,36,38,75,76,77,78,79,80]. In England, the increasing incidence of iGBS disease mainly affects elderly patients, pregnant women and adults with underlying medical conditions, who therefore represent high-risk groups [76]. Similarly, a population-based surveillance study among non-pregnant adults in the United States highlighted an increasing trend of iGBS disease during the period 2008–2016 [77].

The prevalence of GBS serotypes may vary geographically and over time, but different distributions have been demonstrated by age and between colonizing and invasive isolates [26]. In general, unlike neonates and infants, where serotype III tends to predominate [8,81], serotype V, mainly belonging to CC1 and expressing macrolide resistance mediated by the *erm*(B) gene, has emerged since the 1990 and become the most common serotype causing iGBS disease in non-pregnant adults, particularly in North America and Europe [11,12,46,48,82,83,84,85]. A recent study conducted in Brazil reported that serotype V isolates were predominant in invasive infections, while serotypes II and III were more frequent among noninvasive isolates [86].

More recently, however, other serotypes have gained relevance in adult iGBS disease. Serotype III was the most frequent in Norway [85], in Denmark [87], in France [11] and in the restricted area of Brussels-Capital Region, Belgium [88]. Similarly, serotype Ia was the predominant cause of adult iGBS in Belgium [89], Iceland [46], England and Wales [36] and Portugal [30,60]. Serotype II was instead dominant in Ireland [90]. In the US, serotype IV, along with serotypes Ib and II, is assuming an increasingly important role in causing iGBS among non-pregnant adults [38,91].

In Southeast Asia, serotype Ib was the most common serotype in Japan during 2007–2016, followed by serotypes VI and V [92], and in Korea during 2006–2015, followed by III, V, Ia, and VI [93]. Recently, the emergence of an epidemic zoonotic clone (serotype III-4 ST283) associated with the consumption of raw freshwater fish has been described, causing unusually severe and invasive GBS infections in the adult population [94,95].

In this study, serotype III accounted for 75.9% of all iGBS cases, followed by type Ia, and type V. Serotypes Ia, Ib, II, III, IV, and V accounted for all but one of the iGBS isolates (99.1%). A shift from serotype V to serotype III was observed in our country: a previous study on iGBS isolates from non-pregnant adults conducted more than 10 years ago (2002 to 2005) demonstrated that serotypes III and V were equally represented, while in the present study, serotype III showed double the frequency of serotype V [67]. On the contrary, serotype III has always predominated in neonatal and infant invasive GBS infections in our country and Europe [50,96].

This study showed a statistically significant increase in GBS serotype IV in the last period of the survey. The increase in serotype IV is in line with the emergence of this serotype among invasive and colonizing GBS published elsewhere [46,97,98,99]. The serotype IV isolates collected in 2019 belonged to the hypervirulent clone ST1010 (CC452) which possessed HLGR [69]. This finding deserves to be monitored.

All 108 isolates were susceptible to penicillin, but resistance to tetracycline, erythromycin, clindamycin, and high-level gentamicin was found in 85.2%, 26.8%, 24.1% (*n* = 20 cMLS_B_ and 6 iMLC_B_), and 5.5% of isolates, respectively. The first-line antibiotic for both intrapartum antibiotic prophylaxis (IAP) and therapy is a penicillin compound; however, penicillin resistance or non-susceptibility has been increasingly reported recently [44,79,90,100,101,102] and the WHO’s bacterial priority pathogens 2024 list included penicillin-resistant GBS in the medium-priority category. This indicates the need to continuously evaluate its impact on public health, particularly in vulnerable people in resource-limited settings [103]. The penicillin susceptibility of all the GBS isolates in this study confirms the efficacy of this antimicrobial agent as a first-line agent.

Recent studies have reported increasing levels of macrolide and clindamycin resistance in GBS, even though macrolide consumption has significantly decreased in EU/EEA countries over the period 2012–2021 [104], suggesting that the dissemination of particular clones could act as the major driver of this variation [60,75]. These antimicrobials should be used for treatment/IAP only when a penicillin or cephalosporin is not appropriate and after determining the susceptibility of the microorganism. Also, in this study, an increasing trend of erythromycin and clindamycin resistance was observed, reaching the prevalence of 38.1% for both antibiotics in 2019. The prevalences of resistance to erythromycin and clindamycin alone (26.8% and 24.1%, respectively) was in line with those reported from other European countries (about 30%) [60,88]. Outside of Europe, in the same period as our study, resistance to clindamycin reached 43.2% in 2016 in the US [38]. A recent study from Korea reported erythromycin resistance in 33.8% of isolates [93]. In a study from Canada on GBS infections during the years 2014 to 2020, erythromycin resistance increased from 46.4% to 54.0% and clindamycin resistance reached 45% in isolates mainly from adult infections [105]. In light of this, clindamycin resistance may pose a serious challenge to the clinical management of penicillin-allergic patients when used as a second-line agent [36]. By contrast, low prevalences of erythromycin and clindamycin resistance (8.3% and 9.7%, respectively) were found in Iceland [46]. The prevalence of erythromycin resistance found in this study was double that previously reported in Italy (16.5%) [67] but was similar to that found in GBS isolated from invasive neonatal and infants infections (about 28%) isolated in the same years [50]. The cMLS_B_ phenotype with *erm*(B) and the iMLS_B_ with *erm*(A) (subclass *erm*(TR)) (*n* = 6, 20.7%) were the most prevalent phenotypes, in line with what was previously reported [67].

In this study, macrolide resistance was predominant in serotype III (12 isolates), whereas in our previous study, it was mainly due to serotype V isolates. The emerging association of macrolide resistance with serotype III has also been reported elsewhere [11,36,37,67,106,107,108]. By contrast, in a study from Portugal, increasing rates of erythromycin and clindamycin resistance were associated with serotype Ib, with the major driving factor being the expansion of the Ib/CC1 lineage [13]. In Canada, erythromycin resistance increased significantly for serotypes II, III, and V, but capsular type IV, particularly in adult infections, was the capsular type most associated with erythromycin and clindamycin resistance [105].

Tetracycline resistance remained stable during the study period, being found in 92 out of 108 GBS isolates (85.2%); this value is higher than previously reported among adult infections (68.1%), mostly associated with the *tet*(M) gene alone (*n* = 71, 77.2%), as in the past [67].

Resistance to multiple antibiotics in adult iGBS disease is a cause for concern. Inappropriate use of antimicrobials, increasingly older patients with comorbidities, and usage of antibiotics in livestock settings have likely contributed to the increasing antimicrobial resistance [86,109,110]. In this study, 25 (23.1%) iGBS isolates were MDR isolates, resistant to erythromycin, clindamycin and tetracycline, with serotype III being prevalent (11 isolates). Moreover, 7 out of 11 MDR serotype III strains belonged to the hypervirulent MDR-CC17 sublineage that was identified about ten years ago and has been increasingly reported since then [49]. The GBS type III MDR CC-17 sub-clone possesses an integrative and conjugative element (ICE) conferring additional resistance to tetracycline (by *tet*(O) gene), macrolides and lincosamides (*erm*(B) gene), which replaces the genome region encoding pilus 1 [49].

Pili play an important role in bacterial adhesion to epithelial cells and are responsible for colonization, promote biofilm formation, and facilitate translocation across the blood–brain barrier [51,55,56,57,111]. In this study, the most frequent “pilus type” was the combination of PI-1 and PI-2a (44.4%), followed by PI-2a alone (25.9%). In particular, these combinations were present in all the isolates of serotypes Ib, II, and V, in almost all the isolates of serotype Ia, in one-third of serotype III isolates and in almost half of the serotype IV isolates. The combination of PI-1 + PI-2b was found almost exclusively in serotype III (43.9% of all serotype III), and PI-2b alone was observed in capsular type III (17.1% of serotype III) and IV (55.6% of serotype IV isolates). Similar distributions of pili, as well as their associations with the clonal complex/serotype, have been reported in both neonatal and adults populations [73,90,112,113,114,115,116,117].

The surface-anchored protein HvgA is an important adhesin that allows GBS migration into the circulatory and central nervous systems [49,58]. In this study, *hvgA* was found in 26.8% (*n* = 29) of isolates, belonging to only serotypes III (25 isolates) and IV (4 isolates), which presented the PI-2b alone or in combination with PI-1. In other words, in our collection, PI-2b alone was associated with the *hvgA*-positive isolates. PI-2b has been reported to not be limited to only *hvgA*-positive serotype III and IV strains but also found in other clonal lineages and other serotypes, often found not combined with PI-1 [73].

Invasive GBS infection in adults is increasing and is thus responsible for a significant health burden. Vaccination against GBS may offer a solution to reduce morbidity and mortality and antimicrobial use. In recent years, significant progress has been made in vaccine development, primarily to prevent neonatal diseases [118]. GBS have a wide array of surface structures, serving as promising vaccine candidates [119]. A hexa-valent polysaccharide formulation, containing the serotypes Ia, Ib, II, III, IV, and V, which would target almost all neonatal and adult cases, is being evaluated in human clinical trials after encouraging protective effects were demonstrated in animals [120]. The six serotypes accounted for all but one GBS in our adult population, indicating almost complete coverage (99.1%). Although there is no clinical evidence that antibodies can prevent GBS infection in adults, healthy and infected older adults are able to produce anti-GBS CPS antibodies. This has been shown both in vaccine trials and following GBS infection [120]. This study, in line with a previous study, indicated that a vaccine containing the Alp protein family subunits would also have a very high coverage [121,122].

This study has some limitations. First, the present study was based on voluntary reporting, so this surveillance may underestimate the occurrence of iGBS disease in adults. Although cases were reported throughout the country, they may not be indicative of the epidemiological situation at the national level. However, the microbiological investigation carried out provided important data on the serotype distribution, virulence factors, and antibiotic susceptibility trends in adult GBS disease in Italy. Second, the isolates were collected using a hospital-level surveillance system, and clinical information was missing for many patients. For this reason, specific associations between underlying medical conditions, clinical outcomes, length of hospitalization, ward and microbiological data could not be inferred. Finally, this study has been conducted on patients and iGBS strains in the years 2015–2019. Ideally, the data presented in a study should reflect a recent situation; however, in Italy, reporting of human invasive disease by beta-hemolytic streptococci is not mandatory. Most regional reference laboratories send bacterial samples to our laboratory on a semi-annual or annual basis. Therefore, the analysis of the microbiological data of our collection is not conducted in a real-time manner. In particular, we started to receive the bacterial strains from cases that occurred in 2019 after the COVID-19 pandemic period, since 2021. Despite this, we strongly feel that retrospective data can help the scientific community understand whether these constitute geographic niches or represent a true universal trend change underway in the epidemiology of iGBS.

The purpose of this study was not to test the need for changes in current clinical or diagnostic recommendations; our findings reinforce the importance of pursuing continuous surveillance for studying the microbiological epidemiology of iGBS in non-pregnant adults, considering that the elderly population is increasing and paying particular attention to the therapeutic use of macrolides.

## 5. Conclusions

This is the first nationwide study focused on a large sample collection including severe and invasive GBS infections in the adult population. A total of 108 GBS isolates, mostly bacteremic isolates, were analyzed during the period 2015–2019. A higher prevalence of iGBS infections was observed in older adults compared to adults (72 and 32 cases, respectively).

With increasing life expectancy and advances in treatments for complex underlying medical conditions, continued monitoring of the clinical and microbiological characteristics of invasive GBS infections in adults is important. Monitoring the epidemiology of iGBS in adults has highlighted how the characteristics of iGBS disease are evolving. Serotype III is becoming increasingly important compared to serotype V, which was previously the most prevalent serotype in the adult population. An increasing trend of resistance to erythromycin and clindamycin has been observed. Antibiotic resistance, previously predominant in serotype V, is now associated with serotype III. Serotype IV, never detected in the past, is emerging. Penicillin non-susceptibility was not found in Italy, indicating that the use of beta lactams still represents an effective therapeutic approach for GBS infections. This study indicated that the hexa-valent vaccine and a vaccine containing the Alp protein family subunits have a very high coverage in our country.

Continuous surveillance of the GBS pathogenic dynamics, also by the implementation of genomic surveillance, will be essential to develop accurate disease prevention.

## Figures and Tables

**Table 1:** Demographic and clinical data (age, sex, site of isolation, and year of isolation) among adults of 18–64 years and ≥65 years with GBS infections during the period 2015–2019.

Variables, *n* (%)		All ^a^ *n* = 108 ^a^	18–64 Years *n* = 32 ^a^ (30.8%)	≥65 Years *n* = 72 ^a^ (69.2%)	*p* Value	Male *n* = 65	Female *n* = 38	*p* Value
Average age (range)		70.8 (29–97)	50.6 (29–64)	79.7 (65–97)		70.2 (37–95)	70.5 (29–97)	
Sex, *n* (%)	Male	65/103 ^a^ (63.1%)	21/32 (65.6%)	42/69 (60.9%)	0.6463			
	Female	38/103 ^a^ (36.9%)	11/32 (34.4%)	27/69 (39.1%)				
Site of isolation, *n* (%)	Blood	93/104 ^a^ (89.4%)	25/31 (80.6%)	67/72 (93.1%)	0.0614	57/64 (89.1%)	34/37 (91.9%)	0.6465
	Skin and soft tissue	8/104 ^a^ (7.7%)	4/31 (12.9%)	4/72 (5.5%)	0.2013	6/64 (9.4%)	2/37 (5.4%)	n.d.
	Intra-abdominal fluid	2/104 ^a^ (1.9%)	2/31 (6.5%)	0	0.0723	1/64 (1.5%)	1/37 (2.7%)	n.d.
	Bronchial aspirate	1/104 ^a^ (1.0%)	0	1/72 (1.4%)	n.d.	n.a.	n.a.	n.d.
Year, *n* (%)	2015	14/108	4/13 (30.8%)	9/13 (69.2%)	1.0000	11/14 (78.6%)	3/14 (21.4%)	0.1970
	2016	5/108	3/5 (60.0%)	2/5 (40.0%)	0.1466	3/5 (60.0%)	2/5 (40.0%)	0.8827
	2017	46/108	11/45 (24.4%)	34/45 (77.8%)	0.2223	26/43 (60.5%)	17/43 (39.5%)	0.6381
	2018	22/108	7/22 (31.8%)	15/22 (68.2%)	0.9044	9/21 (42.9%)	13/21 (61.9%)	**0.0150**
	2019	21/108	7/19 (36.8%)	12/19 (63.2%)	0.5258	16/19 (84.2%)	3/19 (15.8%)	**0.0348**

Abbreviations: n.d.: not determined; n.a.: not available. For all the comparisons, *p* < 0.05 was considered statistically significant (indicated in boldface). ^a^ Age, sex, and site of isolation of patients with GBS infections were not reported for four, five, and four patients, respectively.

**Table 2:** Clinical manifestations, age group, and sex distributed within the serotypes of 108 iGBS isolates.

	Serotype (*n*, %)							Total (*n*, %)
	Ia	Ib	II	III	IV	V	IX	
Clinical syndrome								
Bacteremia	17 (18.3)	10 (10.7)	5 (5.4)	38 (40.9)	7 (7.5)	15 (16.1)	1 (1.1)	93 (89.4)
SSTI	3 (37.5)	0	0	2 (25)	2 (25)	1 (12.5)	0	8 (7.7)
Intra-abdominal infections	0	0	1 (50)	0	0	1 (50)	0	2 (1.9)
Pneumonia	0	0	0	1 (100)	0	0	0	1 (1.0)
Unknown	0	0	1	0	0	3	0	4
Total	20	10	7	41	9	20	1	108
Age group								
18–64 years	6 (18.75)	2 (6.25)	1 (3.1)	13 (40.6)	4 (12.5)	6 (18.75)	0	32 (30.8)
>65 years	13 (18.1)	8 (11.1)	5 (6.9)	28 (38.9)	5 (6.9)	12 (16.7)	1 (1.4)	72 (69.2)
Unknown	1	0	1	0	0	2	0	4
Total	20	10	7	41	9	20	1	108
Sex								
Male	13 (18.1)	5 (6.9)	4 (5.6)	25 (34.7)	9 (12.5)	9 (12.5)	0	65 (63.1)
Female	7 (18.4)	5 (13.2)	3 (7.9)	15 (39.5)	0	7 (18.4)	1 (2.6)	38 (36.9)
Unknown	0	0	0	1	0	4	0	5
Total	20	10	7	41	9	20	1	108

Abbreviation. SSTI: skin and soft tissue infection.

**Table 3:** Year of isolation, pili, alpha-like surface proteins, *hvgA*, and antimicrobial resistance among different GBS serotypes from adult infections.

Year (*n*, %)	Capsular Type (*n*, %) ^a^							Total (*n* = 108)
	Ia (20, 18.5)	Ib (10, 9.3)	II (7, 6.5)	III ^a^ (41, 38.0)	IV ^a^ (9, 8.3)	V (20, 18.5)	IX (1, 0.9)	
2015	3 (21.4)	3 (21.4)	0	8 (57.1) (**4**) ^a^	0	0	0	14 (13.0)
2016	1 (20.0)	1 (20.0)	1 (20.0)	0	1 (20.0)	1 (20.0)	0	5 (4.6)
2017	11 (23.9)	3 (6.5)	3 (6.5)	15 (32.6) (**8**) ^a^	3 (6.5)	10 (21.7)	1 (2.2)	46 (42.6)
2018	3 (13.6)	2 (9.1)	3 (13.6)	8 (36.4) (**6**) ^a^	1 (4.5) (**1**) ^a^	5 (22.7)	0	22 (20.4)
2019	2 (9.5)	1 (4.8)	0	10 (47.6) (**7**) ^a^	4(19.0) (**3**) ^a^ (*p* **0.0478**) ^c^	4 (19.0)	0	21 (19.4)
Virulence factors (*n*, %)								
PI								
PI-1 + PI-2a	6 (30.0)	6 (60.0)	5 (71.4%)	15 (36.6)	4 (44.4%)	12 (60%)	0	48 (44.4)
PI-1 + PI-2b	2 (10.0)	0	0	18 (43.9) (**18**) ^a^	0	0	0	20 (18.5)
PI-2a	12 (60.0)	4 (40.0)	2 (28.6%)	1 (2.4)	0	8 (40%)	1 (100%)	28 (25.9)
PI-2b	0	0	0	7 (17.1) (**7**) ^a^	5 (55.6%) (**4**) ^a^	0	0	12 (11.1)
hvgA	0	0	0	25 (61.0)	4 (44.4%)	0	0	29 (26.8)
alpha-like surface protein ^b^ (*n*, %)								
alpha C	4 (20.0)	10 (100.0)	2 (28.6)	3 (7.3)	5 (55.6)	6 (30.0)	1 (100)	31 (28.7)
alp1	12 (60.0)	0	0	2 (4.9)	3 (33.3)	5 (25.0)	0	22 (20.4)
alp 2/3	3 (15.0)	0	1 (14.3)	0	1 (11.1)	7 (35.0)	0	12 (11.1)
rib	1 (5.0)	0	4 (57.1)	35 (85.4)	0	1 (5.0)	0	41 (38.0)
neg	0	0	0	1 (2.4)	0	1 (5.0)	0	2 (1.8)
Antimicrobial resistance								
Erythromycin (*n*, %)	3 (15.0)	4 (40.0)	0	12 (29.3)	2 (22.2)	8 (40.0)	0	29 (26.8)
Clindamycin (*n*, %)	1 (5.0)	4 (40.0)	0	11 (26.8)	2 (22.2)	8 (40.0)	0	26 (24.1)
Tetracycline (*n*, %)	19 (95.0)	7 (35.0)	7 (100%)	38 (92.7)	4 (44.4)	17 (85.0)	0	92 (85.2)
HLGR (*n*, %)	0	1 (5.0)	0	0	4 (44.4)	1 (5.0)	0	6 (5.5)
Macrolide resistance phenotype (*n*)	CR(1); M(2)	CR(4)	0	CR(9); IR (2); M(1)	CR(2)	CR(4); IR(4)	0	CR(20); IR(6); M(3)
Macrolide resistance genes (*n*)	*erm*B(1); *mef*A/E(2)	*erm*B(4)		*erm*B(9); *erm*A(2); *mef*A/E(1)	*erm*B(2)	*erm*B(4); *erm*A(4)	-	*erm*B(20); *erm*A(6); *mef*A/E(3)
Tetracycline resistance genes (*n*)	*tetM*(18); *tetM* + *tetO*(1)	*tetM*(3); *tetO*(4)	*tetM*(6); *tetM* + *tetO*(1)	*tetM*(25); *tetO*(11); *tetM* + *tetO*(1); not detected(1)	*tetM*(2); *tetO*(1); *tetM* + *tetO*(1)	*tetM*(17)	-	*tetM*(71); *tetO*(16); *tetM* + *tetO*(4); not detected(1)
MDR ^b^ (*n*)	*erm*B/ *tetM*(1)	*erm*B/ *tetO*(4)	0	*erm*B/*tetO*(7); *erm*B/*tetM*(2); *erm*A/*tetM*(2)	*erm*B/*tetM*(1); *erm*B/*tetM + tetO*(1)	*erm*B/*tetM*(4); *erm*A/*tetM*(3)	0	*25*

Abbreviations: CR: constitutive MLS_B_ (macrolide–lincosamide–streptogramin B) resistance phenotype (cMLS_B_); IR: inducible MLS_B_ resistance phenotype (iMLS_B_); M: macrolide resistance phenotype (M); PI: pilus island; HLGR: high-level gentamycin resistance; PI: pilus island. ^a^ The number of *hvgA* gene-positive GBS isolates is indicated in parenthesis in boldface (***n***). ^b^ MDR isolates defined a resistance to ≥3 antimicrobial agents of different classes [74]. All the MDR isolates showed resistance to tetracycline, erythromycin, and inducible or constitutive clindamycin resistance. One serotype Ib MDR isolate was HLGR. ^c^ For all the comparisons, *p* < 0.05 was considered statistically significant (indicated in boldface and underscored). The association between the year of isolation 2019 and serotype IV was statistically significant.

**Table 4:** Description of 25 *hvgA*-serotypes III isolates inferred to belong to the hypervirulent serotype III/ST17 clone using specific PCR.

	Adults (18–64 yrs) (*n*, %)	Older Adults (≥65 yrs) (*n*, %)	Year of Isolation (*n*, %)	Site of Isolation (*n*, %)	Alpha-like/*hvgA* (*n*, %)	Pilus Island (*n*, %)	MDR (*n*, %)	Erythromycin Resistance Genes (*n*, %)	Tetracycline Resistance (*n*, %)	Tetracycline Resistance Genes (*n*, %)
25 isolates (23.1%)	8, 32.0%	17, 68.0%	2015 (4, 16.0)); 2017 (8, 32.0%); 2018 (6, 24.0%); 2019 (7, 28.0%)	Blood (25, 100.0%)	*rib*/*hvgA* (25, 100.0%)	PI-1 + PI-2b (18, 72.0%); PI-2b (7, 28.0%)	7, 28.0%	*ermB* (9, 100.0%)	25, 100.0%	*tetM* (16, 64.0%); *tetO* (7, 28.0%); *tetM* + tetO (1, 4.0%); nd (1, 4.0%)

Abbreviations. Yrs: years; nd: *tetM* and *tetO* genes not detected.

**Table 5:** Prevalence of erythromycin, clindamycin, and tetracycline resistance and associated resistance genes over time.

Year (*n*)	Resistance to			Resistance Genes	
	Ery (*n*, %)	Cli (*n*, %)	Tet (*n*, %)	Ery (*n*)	Tet (*n*)
2015 (14)	2 (14.3)	0 (0.0)	11 (78.6)	*mefA* (2)	*tetM* (10); *tetO* (3)
2016 (5)	1 (20.0)	1 (20.0)	5 (100.0)	*ermB* (1)	*tetM* (4); *tetO* (1)
2017 (46)	12 (26.1)	11 (23.9)	42 (91.3)	*ermB* (8); *ermA* (3); *mefA* (1)	*tetM* (35); *tetO* (6)
2018 (22)	6 (27.3)	6 (27.3)	18 (81.8)	*ermB* (6)	*tetM* (14); *tetO* (5)
2019 (21)	8 (38.1)	8 (38.1)	16 (76.2)	*ermB* (5); *ermA* (3)	*tetM* (12); *tetO* (5)

Abbreviation. Ery: erythromycin; Cli: clindamycin; Tet: tetracycline; *n*: number of isolates.

## Data Availability

The original contributions presented in the study are included in the article. Further inquiries can be directed to the corresponding author.
